# Polyarteritis nodosa in childhood - expertise of a rheumatology pediatric unit

**DOI:** 10.1186/1546-0096-12-S1-P362

**Published:** 2014-09-17

**Authors:** Joana Abelha-Aleixo, Mariana Rodrigues, Francisca Aguiar, João Barreira, Iva Brito

**Affiliations:** 1 Rheumatology, Centro Hospitalar de São João, Porto, Portugal; 2 Pediatric, Centro Hospitalar de São João, Porto, Portugal; 3 Rheumatology Pediatric Unit, Centro Hospitalar de São João, Porto, Portugal

## Introduction

Polyarteritis nodosa (PAN) is the 3th most common chilhood vasculitis, although rare (3% of cases).

## Objectives

The authors intend to describe the PAN cases in our Rheumatology pediatric unit (RPU)

## Methods

Retrospect review of the medical records of the PAN patients of our RPU.

## Results

### Case 1

A 2 year old boy with Klinefelter syndrome hospitalized for ataxia and ocular deviation due to stroke. Deficits resolved within a week. Six months later he was readmitted with left hemiparesis. Brain MRI revealed an ischemic thalamic lesion.Prothrombotic study was negative. At 4 years he was admitted for fever,rash,arthralgia and Raynaud's phenomenon.Infectious causes were excluded and autoimmune study was negative. At 6 years he was hospitalized for hypertension with preserved renal function. CT scan showed renal cortical lesions. Three months later he developed flaccid paresis of the right leg with sphincters incontinence. The study revealed spinal cord ischemic injury and multiple renal microaneurysms.PAN was diagnosed, treated with oral prednisolone (PDN) 1mg/kg/day, and 13 cycles of intravenous cyclophosphamide (CYC).Currently he is 10 years, under PDN 5mg/d,warfarin and aspirin and on clinical remission.

### Case 2

Girl with 8 months old hospitalized for fever,vomiting and diarrhea with left ptosis and paresis of the left arm. She also had hypertension,normochromic/normocytic anemia,and slight elevation of ESR and CRP.Lumbar puncture and cultures were negative and brain MRI showed ring-shapped lesions suggestive of abcess.Broad-spectrum antibiotic therapy was started, with improvement,but then she developed 2 episodes of intestinal occlusion needing surgery. Histology was compatible with vasculitis of small/medium-sized arteries with fibrinoid necrosis and inflammatory infiltrates. The autoimmune study was negative.PAN was diagnosed, treated with pulses of methylprednisolone followed by oral PDN with clinical amelioration.Six months later she developed left hemiparesis with III cranial nerve palsy. She held 6 cycles of CYC followed by azathioprine. Currently she is 5 years, under aspirin 100mg/d and ramipril 5mg/day. Immunossupression was stopped 2 years ago maintaining clinical remission.

### Case 3

A nine year old boy hospitalized for fever,headache and persistent vomits. He was hypotensive, had a systolic murmur and diffuse abdominal pain. The workup study showed leukocytosis, elevated CRP (286mg/L),renal insufficiency, negative autoimmune antibodies, bilateral pleural effusion, hepatosplenomegaly and proteinuria. An exploratory laparotomy revealed mesenteric adenitis.Renal biopsy demonstrated vasculitic lesions of small/medium-sized arteries suggestive of PAN.He needed invasive ventilation and initiated PDN 1mg/kg/d plus CYC,with clinical amelioration. Then anisocoria and right hemiparesis were noticed caused by and isquemic stroke with extensive hemorrhagic transformation. Urgent decompression surgery was made.He had slow clinical recovery with physical rehabilitation and underwent 12 cycles of CYC followed by azathioprine. PDN was slowly tapered. He remained with vascular epilepsy and mild right hemiparesis. Today he is 12 years old on clinical remission without PDN.

## Conclusion

We described three PAN cases all with very agressive presentations, central nervous system involvement and ANCA negative, with relatively good response to immunossupression. Only one remained with sequelae, but all have currently good organic and cognitive prognosis and no disease relapses.

## Disclosure of interest

None declared.

